# Effects of upper extremity surgery on activities and participation of children with cerebral palsy: a systematic review

**DOI:** 10.1111/dmcn.14315

**Published:** 2019-07-23

**Authors:** Annoek Louwers, Jessica Warnink‐Kavelaars, Joost Daams, Anita Beelen

**Affiliations:** ^1^ Department of Rehabilitation, Amsterdam UMC University of Amsterdam Amsterdam the Netherlands; ^2^ Medical Library, Amsterdam UMC University of Amsterdam Amsterdam the Netherlands; ^3^ Center of Excellence in Rehabilitation Medicine UMC Utrecht Brain Center, University Medical Center Utrecht, De Hoogstraat Rehabilitation Utrecht the Netherlands; ^4^ Department of Rehabilitation, Physical Therapy Science and Sports UMC Utrecht Brain Center, University Medical Center Utrecht the Netherlands

## Abstract

**Aim:**

To evaluate and synthesize the evidence for effects of upper extremity surgery (UES) on activities and participation of children and adolescents with cerebral palsy (CP).

**Method:**

The databases MEDLINE, Embase, and PsycINFO were searched for publications up to September 2018. Studies included were comparative studies with or without concurrent comparison groups or case series with pretest/posttest outcomes with a minimal sample size of 10 participants; those that reported the effects of UES with a follow‐up time of at least 5 months; those including patients diagnosed with CP aged up to 20 years; and those that used a validated activity‐based instrument. Risk of bias was assessed using the ROBINS‐I (Risk Of Bias In Non‐randomised Studies ‐ of Interventions) tool and quality assessment was performed using the Grading of Recommendations Assessment, Development and Evaluation.

**Results:**

Twelve studies, involving 310 children and adolescents, were included. The ability and perception of the patient to use the hand(s) and perform activities (measured with the Shriners Hospital Upper Extremity Evaluation, Assisting Hand Assessment, and House Functional Classification) improved significantly after UES. The quality of evidence was very low for each of the activity outcomes of interest.

**Interpretation:**

The very low evidence prohibits recommendations on the use of UES to guide clinical practice. More high‐quality comparative studies are needed to obtain better insight into the effects of UES on activities and participation.

**What this paper adds:**

- Low quality of evidence for effects of upper extremity surgery (UES) on activities and participation.
- Limited evidence for improvement in activities and participation after UES.

## What this paper adds

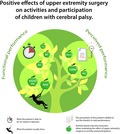

This article's abstract has been translated into Spanish and Portuguese.

Follow the links from the http://onlinelibrary.wiley.com/doi/10.1111/dmcn.14315/abstract to view the translations.

## Resumen

**Objetivo:**

Evaluar y sintetizar la evidencia de los efectos de la cirugía de extremidades superiores (UES) sobre las actividades y la participación de niños y adolescentes con parálisis cerebral (PC).

**Método:**

En las bases de datos MEDLINE, Embase y PsycINFO se buscaron publicaciones hasta septiembre de 2018. Los estudios incluidos fueron estudios comparativos con o sin grupos de comparación concurrentes o series de casos con resultados de prueba previa / prueba posterior con un tamaño de muestra mínimo de 10 participantes; aquellos que informaron los efectos de UES con un tiempo de seguimiento de al menos 5 meses; aquellos que incluyen pacientes diagnosticados con PC de hasta 20 años; y aquellos que utilizaron un instrumento validado basado en actividades. El riesgo de sesgo se evaluó mediante la herramienta ROBINS‐I (Riesgo de sesgo en estudios no aleatorios ‐ Intervenciones) y la evaluación de la calidad se realizó mediante la evaluación, desarrollo y evaluación de la calificación de las recomendaciones.

**Resultados:**

Se incluyeron 12 estudios con 310 niños y adolescentes. La capacidad y la percepción del paciente para usar la/s mano/s, y realizar las actividades (medidas con la Evaluación de la extremidad superior del Hospital Shriners, la Evaluación de la mano auxiliar y la Clasificación funcional de la casa) mejoraron significativamente después de la UES. La calidad de la evidencia fue muy baja para cada uno de los resultados de actividad de interés.

**Interpretación:**

La evidencia muy baja previene realizar recomendaciones sobre el uso de UES para guiar la práctica clínica. Se necesitan más estudios comparativos de alta calidad para obtener una mejor comprensión de los efectos de UES en las actividades y la participación.

## Resumo

**Objetivo:**

Avaliar e sintetizar a evidência dos efeitos de cirurgia do membro superior (CMS) sobre as atividades e participação de crianças com paralisia cerebral (PC).

**Método:**

As bases de dados MEDLINE, Embase, e PsycINFO foram pesquisadas quanto a publicações até setembro de 2018. Foram incluídos estudos comparativos com ou sem grupos de comparação concorrentes ou séries de casos com resultados pré/pós‐teste com um tamanho amostral mínimo de 10 participantes; aqueles que reportaram os efeitos de CMS com tempo de acompanhamento de pelo menos 5 meses; aqueles incluindo pacientes com diagnóstico de PC e até 20 anos de idade; e aqueles que usaram um instrumento válido para avaliar atividade. O risco de viés foi avaliado usando o instrumento RVENA‐I (Risco de viés em estudos não aleatorizados – de intervenções) e a avaliação da qualidade foi realizada com a Pontuação de Recomendações, Avaliação, Desenvolvimento e Mensuração.

**Resultados:**

Doze estudos, envolvendo 310 crianças e adolescentes, foram incluídos. A capacidade e percepção do paciente em utilizar as mãos e realizar atividades (mensuradas com a Avaliação do Hospital Shriners para a extremidade superior, a Avaliação da Mão Auxiliar, e a Classificação Funcional de House) melhoraram significantemente após CMS. A qualidade da evidência foi muito baixa para os resultados de atividade de interesse.

**Interpretação:**

A evidência muito baixa proíbe recomendações sobre o uso de CMS para guiar a prática clínica. Mais estudos comparativos de alta qualidade são necessários para obter mais informações a respeito dos efeitos de CMS nas atividades e participação.

**Abbreviations**
AHA: Assisting Hand Assessment
BBT: Box and Block Test
COPM: Canadian Occupational Performance Measure
JHFT: Jebsen–Taylor Hand Function Test
SHUEE: Shriners Hospital Upper Extremity Evaluation
UES: Upper extremity surgery

Children and adolescents with cerebral palsy (CP) represent the largest diagnostic group treated in paediatric rehabilitation. The upper limbs are often affected with significant wrist and hand involvement.^1^ The abnormal upper limb tone and hand posture, most frequently forearm pronation, thumb adduction, and/or flexion with limited wrist extension, have an impact on the ability to use the affected hand and both hands together.^2^

One of the treatment options for improving functional performance is upper extremity surgery (UES). Unfortunately, there is a substantial emphasis in the medical literature reporting outcomes on the effect of UES relating to the ‘body functions and structures' component of the International Classification of Functioning, Disability and Health for Children and Youth:^3^ i.e. motor outcomes, active and passive range of motion and spasticity reduction, position of the hand, appearance, and grip function.^4^ Less clear and less reported is the effect of UES on the International Classification of Functioning, Disability and Health for Children and Youth component of ‘activities and participation'.^3^

UES aims to improve muscle balance and hand posture, by releasing or lengthening spastic muscles, transferring tendons, and stabilizing joints. Commonly used procedures are: (1) release of pronator teres muscle to facilitate forearm supination;^5^, ^6^ (2) release or transfer of the flexor carpi ulnaris tendon to facilitate or increase wrist extension for functional grip purposes;^7^ and (3) correction of a thumb‐in‐palm deformity, preferably by adductor pollicis muscle slide^8^ combined with extensor pollicis longus rerouting.^9^ In contrast to activity‐based interventions, UES is an invasive intervention followed by immobilization in plaster for 5 to 6 weeks and then an intensive therapy programme, depending on the goals and type of surgery.

Over the past decade, more studies have used validated activity‐based instruments to evaluate the effect of UES on activities: i.e. the use of the affected hand and perceived performance. The execution of activities can be assessed according to the patient's functional performance (what the patient usually does), by testing what a patient is able to do on request (capacity), or by evaluating the patient's self‐perception of the ability to use the affected arm. An overview of evidence about the effect of UES on activities and participation is needed to guide medical professionals in intervention planning and to inform patients on the risks, benefits, and possible outcomes of UES on daily activities.

The aim of this systematic review was to evaluate and synthesize the evidence for the effects of UES on activities and participation in activities of children and adolescents (<20y)^3^ with CP. This review will focus on two types of activity‐based outcome measure: (1) functional performance outcome measures (clinical‐based), which reflect the patient's performance on quantifiable tasks, classification, or questionnaire; and (2) perceived performance outcome measures (patient‐reported), which reflect the perception of the patient's ability to use the hand(s) in task performance.

## Method

This systematic review was conducted using the Preferred Reporting Items for Systematic Reviews and Meta‐Analyses (PRISMA) guidelines.^10^ The review has been registered in the international prospective register of systematic reviews (PROSPERO CRD42017058753).

### Eligibility criteria

Published studies investigating the effect of UES in children and adolescents with CP were included if they met the following criteria: (1) They were (pseudo‐)randomized controlled trials, comparative studies with concurrent comparison groups (including a non‐randomized experimental trial, prospective and retrospective cohort study, case–control study, and interrupted time series with a comparison group), comparative studies without comparative individuals (including a historical control study, two or more single‐arm studies, and interrupted time series without a parallel comparison group), or case series with either posttest or pretest/posttest outcomes with a minimal sample size of 10 participants (because smaller studies are associated with a very high risk of [selection] bias); (2) A validated activity‐based instrument was used to measure the effect of UES on activities and participation; (3) The UES intervention was not combined with botulinum neurotoxin A injections 6 months before surgery; and (4) More than 75% of the participants were children or adolescents diagnosed with CP aged up to 20 years or, if data on the children/adolescents with CP were reported separately, to ensure that study results were generalizable to CP. Studies were excluded if no full‐text was available in English.

### Search strategy

The comprehensive literature search started with a reference set collected through citation analysis (cited by tracking) in Google Scholar. References in the included articles were checked and used to complete the reference set up to 45 articles. This reference set was used to derive the following major search concept combination: [cerebral palsy] AND ([releasing/lengthening] OR {[surgery/therapy] AND [anatomic region]}).

The literature search was conducted in three electronic databases indexing health‐related journals, for publication up to September 2018: MEDLINE, Embase, and PsycINFO.

The search strategy was adapted to match the controlled vocabulary and search syntax of each database (see Appendix S1 [online supporting information] for details). The search strategy was developed to maximize the sensitivity of article identification. All items in the reference set had to be retrieved by the systematic search strategy in all databases if present.

Review authors also searched reference lists of included studies and other narrative reviews, identifying no additional reference titles. All duplicates were removed. The search strategy was designed in collaboration with a clinical librarian according to the recommendations of the PRISMA guidelines.^10^

### Study selection

Titles and abstracts were initially screened for study eligibility/relevance by the first author (AL) with the last author (AB) screening excluded titles and abstracts to ensure that no relevant papers were omitted. After the initial identification and screening, full‐text articles reporting the effect of UES in children/adolescents with CP were reviewed and independently assessed for eligibility according to the inclusion and exclusion criteria by two reviewers (AL and AB). Cases of disagreement were discussed until consensus was reached.

### Data extraction

Two reviewers (AL and AB) extracted information from each included study independently and reported their findings on a data extraction sheet on the basis of the Oxford Centre for Evidence‐Based Medicine Scale V2.1.^11^ Disagreements were resolved by discussion between the two review authors. No authors were contacted to provide additional data.

Information was extracted from each included study on (1) study design; (2) characteristics of the study sample (including age, types of CP); (3) surgical (contra‐)indications; (4) surgical technique; (5) length of follow‐up; (6) comparative subgroups; and (7) types of activity‐based outcome measure.

### Risk of bias in individual studies

The risk of bias for non‐randomized observational interventional studies with validated outcome measures was evaluated by two reviewers (AL and AB) working independently using the ROBINS‐I (Risk Of Bias In Non‐randomised Studies ‐ of Interventions) measurement tool.^12^ Each article was assessed as having low, high, or unclear risk for six criteria and is reported in the tables of characteristics of included studies (Table SI, online supporting information) and risk of bias (Table 1).

**Table 1:** ROBINS‐I risk of bias assessment

Study	Before intervention		At intervention	After intervention				Overall risk of bias assessment
	Confounding	Selection of participants	Classification of intervention	Deviation from intended interventions	Missing data	Measurement of outcomes	Selection of reported result	
Carlson et al.^20^	C	C	S	C	S	S	S	C
Donadio et al.^22^	C	C	S	C	S	S	S	C
House et al.^23^	C	C	S	C	S	S	M	C
Libberecht et al.^18^	C	C	S	C	S	M	M	C
Louwers et al.^15^	S	M	S	S	L	M	M	S
Matsou et al.^24^	C	C	S	C	S	S	S	C
Matsou et al.^25^	C	C	S	C	S	S	S	C
Ponten et al.^16^	C	S	S	S	L	S	M	C
Roth et al.^21^	C	C	S	C	S	S	S	C
Smitherman et al.^17^	S	M	S	S	M	M	M	S
Van Heest et al.^19^	C	C	S	S	S	M	M	C
Van Heest et al.^14^	S	S	L	L	M	M	M	S

Each domain is determined to exhibit low (L), moderate (M), serious (S), or critical (C) risk of bias. Low risk of bias indicates that the study is ‘comparable to a well‐performed randomized trial' in the domain being evaluated. Moderate risk of bias indicates the study is ‘sound for a non‐randomized study' but not comparable to a rigorous randomized trial. Serious risk of bias indicates the presence of ‘important problems'. Critical risk of bias indicates the study is ‘too problematic to provide any useful evidence on the effects of intervention'. The overall risk of bias of each study was equal to the most severe level of bias found of any domain. ROBINS‐I, Risk Of Bias In Non‐randomised Studies ‐ of Interventions.

### Assessment of the quality of evidence

The quality of the body of evidence for each outcome was examined by two assessors (AB and AL) according to the Grading of Recommendations Assessment, Development and Evaluation (GRADE) Working Group criteria.^13^ GRADE was used to summarize the body of evidence and to enable quicker interpretation of the findings of the review for clinical practice. The overall quality of evidence was determined to be high, moderate, low, or very low using a stepwise, structural methodology. This was done for outcomes of interest across the studies, as well as for the strength of recommendation for using the UES intervention. The scores were compared and disagreements were resolved by discussion and consensus.

### Data synthesis

Given the heterogeneity of UES interventions (within and between studies) and reported outcomes on activities and participation, a meta‐analysis could not be performed. Therefore a narrative synthesis of results is presented.

## Results

### Study selection

The electronic search provided a total of 8552 citations. After adjusting for duplicates, 6501 remained. Of these, 6366 studies were discarded because, after reviewing the abstracts, it seemed that these papers were not published with a full text in English (*n*=603) or did not meet the criteria (*n*=5763). The full text of the remaining 140 citations was examined in more detail. One hundred and fourteen studies did not meet the inclusion criteria as described, mainly because of a different outcome or population and small numbers of participants. Fourteen studies were excluded because data were not presented separately or fewer than 75% of the participants were patients diagnosed with CP aged up to 20 years. Finally, a total of 12 studies were identified for inclusion in the review (Fig. S1, online supporting information).

#### Characteristics of included studies

A summary of the included studies is presented in Table SI. The three prospective studies stated measurement time points in advance^14^, ^15^, ^16^ and two retrospective studies described the use of a standard protocol which included follow‐up time‐points.^17^, ^18^ Five studies provided information about missing data and loss to follow‐up (Table SI).^14^, ^15^, ^17^, ^18^, ^19^ Ten studies included only children and adolescents with CP aged up to 24 years.^14^, ^15^, ^16^, ^17^, ^19^, ^20^, ^21^, ^22^, ^23^, ^24^ From the two studies including patients older than 20 years of age,^18^, ^25^ the percentage patients to include (>75% of the participants were aged up to 20y) could be derived from the reported mean and range. Four studies included participants with unilateral CP,^14^, ^17^, ^19^, ^21^ of which three involved those described as having spastic CP.^17^, ^19^, ^21^ Indications were based on presentation of pronation deformity, deficient active wrist extension with adequate digital control, wrist flexion deformity with the flexor carpi ulnaris as the primary deforming force, and adduction of the thumb ray with flexion at the thumb.

### Risk of bias within studies

There was an overall high risk of bias within the included studies (Table 1). In all retrospective studies the risk of bias was critical for the items on selection (owing to lack of allocation concealment, loss to follow‐up), confounding (inherently to non‐controllable studies, different baseline characteristics), and measurement of outcomes (blinding of outcome assessment). Three studies^14^, ^15^, ^17^ were judged to be at serious risk of bias in at least one domain, but not at critical risk of bias in any domain.

### GRADE assessment of the quality of evidence

The quality of evidence was very low for each postoperative activity‐based outcome after UES in children and adolescents with CP (Table 2). The scarcity of comparative studies, the selection of participants, and the lack of blinding in outcome assessment introduced a high risk of bias, which was accounted for in the GRADE analysis.

### Activity‐based outcome measures

Table 3 presents the activity‐based outcome measures used to evaluate the effect of UES on functional and perceived performance. Clinicians evaluated patients' functional performance using an outcome measure including quantifiable tasks in 12 studies.^14^, ^15^, ^16^, ^17^, ^18^, ^19^, ^20^, ^21^, ^22^, ^23^, ^24^, ^25^ The perception of patients' abilities to use the hand(s) in task performance was measured with the Canadian Occupational Performance Measure (COPM) in three studies.^14^, ^15^, ^16^

#### Functional performance outcome measures (clinical‐based)

Patients' abilities to handle objects on request (capacity) was evaluated with the Box and Block Test (BBT),^26^ the Jebsen–Taylor Hand Function Test (JHFT),^27^ and the House Functional Classification^23^ in seven studies,^18^, ^20^, ^21^, ^22^, ^23^, ^24^, ^25^ ranging from 0 (no use of the hand) to 8 (complete spontaneous use of the hand). The mean House Functional Classification score changed significantly in the studies, suggesting that the affected arm/hand changed from a (fair) passive assist before surgery to a (fair) active assist after surgery.^23^ Scoring of the JHFT and the BBT is based on the time it takes the patient to perform the unimanual tasks. No change after UES was found in the speed of performing a unimanual task performed with the affected hand. For interpretation of change, knowledge about the minimal clinically important difference is important. Unfortunately, these differences are not known for the JHFT, BBT, and House Functional Classification.

Patients' functional performance of activities was evaluated with the Assisting Hand Assessment (AHA)^28^, ^29^, ^30^, ^31^ and the Shriners Hospital Upper Extremity Evaluation (SHUEE) spontaneous functional analysis.^32^ Significant improvement on the spontaneous functional use (SHUEE spontaneous functional analysis and AHA) was found in all four studies.^14^, ^15^, ^16^, ^17^ For the AHA, a change of five AHA units or more does (with 95% certainty) reflect a real change exceeding any random measurement error.^33^

#### Perceived performance outcome measures (patient‐reported)

Three studies used the COPM^34^, ^35^, ^36^, ^37^ to explore the perception of the patients' abilities to use the hand(s) in task performance,^14^, ^15^, ^16^ but only one study used the COPM to measure change after UES.^15^ The mean COPM score in this study showed a significant and clinical relevant change (≥2 points) on patients' perceptions of the performance and satisfaction of selected goals after UES. The other two studies presented the outcomes by using one part of the COPM (performance); these showed an improved performance of selected goals from 2.6 (range 1–8) to 6.4 (3–10),^16^ or did not report the change in COPM performance for the surgical intervention group separately but reported no difference between the three intervention groups.^14^

## Discussion

This systematic review has shown that there is very low quality of evidence for the effects of UES on activities and participation of children and adolescents with CP. The methodological quality of the 12 included studies was poor and evidence for comparative effects is limited.

Previous reviews have demonstrated positive effects of UES in children and adolescents with CP on upper limb function (i.e. dexterity, range of motion at the wrist and forearm, and grip strength).^38^, ^39^, ^40^ Our review evaluated the effect on patients' functional and perceived performance measured with validated activity‐based outcome measurements rated by clinicians or patients/caregivers. Patients' functional performances were measured in five studies^14^, ^15^, ^16^, ^17^, ^19^ on quantifiable tasks with the SHUEE, AHA, JHFT, or BBT and in seven studies^17^, ^18^, ^21^, ^22^, ^23^, ^24^, ^25^ by a classification.

After UES, improvements were reported in patients' functional performance (what the patient usually does) (SHUEE, AHA, and ABILHAND) and on patients' self‐perception of the ability to use the affected arm (COPM). No improvements were noted for speed of hand skills or what a patient is able to do on request (capacity) (JHFT and BBT). However, interpreting these findings is limited in view of the methodological limitations of the studies.

### Implications for practice and research

Considering the overall very low quality of the evidence, it is not possible to make clinical recommendations on the effect of UES. However, the positive results of the effect of UES on activities and participation presented in all included studies certainly justify future controlled studies.

#### A core set of validated outcome measures

Different validated activity‐based outcome measures were used in the included studies, which showed that clinicians and patient/caregivers were positive about the effect on activities and participation after UES. To enhance the comparability of studies and to perform pooled analysis, the use of a core set of validated outcome measures to gather and share data in international databases is recommended. This information will be important to improve selection criteria, to indicate specific surgical procedures, and to predict outcomes for UES.

On the basis of the findings of this review, we suggest adding at least the three activity‐based outcome measures to the core set when evaluating the effect of UES on activity and participation: the AHA, SHUEE, and COPM. The SHUEE and AHA should be included because they evaluate the use of both hands together; the SHUEE has more components of measuring capacity and the AHA of measuring performance. The COPM can be used to measure patient‐related outcomes of perceived performance and satisfaction of the ability to perform the activity.

Apart from these activity‐based outcome measures, more assessments at all levels of the International Classification of Functioning, Disability and Health (‘body function and structure', ‘activity and participation') are needed to recognize the impact of the changes after UES on patients' functioning and the possibilities for engaging fully in their lives.

#### Effect of postoperative therapies after UES

To be able to interpret the effect of UES it is necessary to have consensus about postoperative therapy, because the use of different therapies is likely to influence the outcomes after UES on activites and participation. Future studies should compare postoperative therapies to identify the optimal frequency, duration, intensity, and focus (to improve function and/or activities and participation). A period of postoperative immobilization in plaster will always follow UES. However, there is a lack of knowledge about the effect of immobilization in plaster on the results after UES. More knowledge is also needed about aspects such as the optimal position of the arm/hand (e.g. the degree of any over‐correction) and the duration of casting.

#### Long‐term outcomes after UES

Future studies should also evaluate long‐term outcomes. It is not well known whether UES interventions continue to add benefit over years or whether the gains are lost with ageing. More high‐quality comparative effect studies with a large number of participants are needed to determine optimal patient selection criteria and the indications for specific UES procedures.

### Limitations

The findings of this systematic review should be interpreted in the context of its limitations. Furthermore, the retrospective case‐series design used in most studies means the evidence is initially rated low, and has a high risk of bias, such as recall and publication bias which may have limited the available evidence.

Many studies were excluded because they used unique, individualized, and often qualitative activity‐based outcome measures. For example, daily activities were evaluated as a qualitative measure of function, or a questionnaire was used in which participants were asked about the change in hand use after UES. A lack of reported statistical precision (e.g. no estimates of effects with confidence intervals) was found within the included studies, making pooled analyses impossible.

## Conclusions

This systematic review has shown that there is very low quality of evidence for the effects of UES on activities and participation of children and adolescents with CP. In all 12 included studies, the ability and perception of the patient to use the hand(s) and perform activities improved significantly after UES. However, studies had poor methodological quality and reported very low quality of evidence for the effects of UES on activities and participation of children and adolescents with CP. More high‐quality multidisciplinary studies are needed to obtain better insight into the comparative effects of UES on activities and participation in this group.

**Table 2:** Outcomes of upper extremity surgery on activities and participation of children and adolescents with cerebral palsy

Outcome measure	Participants (study)	Mean change in score (SD)	95% CI of the mean	*p* (provided by author)	Quality of evidence^a^
JHFT					Very low
	13^19^	—	—	0.81	
BBT					Very low
	16^14^	2.0 (5.9)	−1.85 to 4.65	>0.05	
	27^15^	2.3 (4.9)	0.36–4.13	0.21	
HFC					Very low
	24^20^ 8^20^	2.0 (2.0) 2.0 (2.0)	0–2.0 0–4.0	<0.01 <0.01	
	20^22^	1.95 (1.4)	1.32–2.58	<0.001	
	56^23^	2.7 (1.7)	0.22–2.23	<0.001	
	15^18^	3.0 (—)	—	0.002	
	26^25^	1.7 (1.7)	0.98–2.35	<0.001	
	19^24^	3.2 (1.2)	2.59–3.70	<0.001	
	17^21^	1.8 (—)	—	<0.001	
SHUEE SFA					Very low
	40^17^	4.0 (—)	—	<0.001	
	16^14^	6.5 (11.0)	0.37–12.56	<0.05	
AHA					Very low
	18^16^	6.2 (3.7)	4.34–8.0	<0.001	
	16^14^	3.1 (5.4)	0.21–5.92	<0.05	
	31^15^	6.7 (4.2)	5.14–8.21	<0.001	
COPM‐P					Very low
	35^15^	3.2 (1.6)	2.58–3.72	<0.001	
COPM‐S					Very low
	35^15^	3.3 (2.1)	2.55–3.99	<0.001	
ABILHAND					Very low
	34^15^	1.5 (1.2)	1.08–1.93	<0.001	
VAS					Very low
	29^15^	2.4 (1.9)	1.66–3.14	<0.001	

a Grading of Recommendations Assessment, Development and Evaluation.

JHFT, Jebsen–Taylor Hand Function Test; BBT, Box and Block Test; HFC, House Functional Classification; SHUEE SFA, Shriners Hospital Upper Extremity Evaluation spontaneous functional analysis; AHA, Assisting Hand Assessment; COPM‐P, Canadian Occupational Performance Measure performance scale; COPM‐S, Canadian Occupational Performance Measure satisfaction scale; VAS, visual analogue scale.

**Table 3:** Activity‐based outcome measures evaluating the effect of upper extremity surgery. (a) Measuring functional performance (clinical‐based). (b) Measuring perceived performance (patient‐reported)

(a)			
Outcome measures	Study	Improvement	MCID (% of participants)
Quantifiable tasks			
Measuring capacity			
Box and Block Test^26^	Van Heest et al.^14^	Not significant	Unknown^a^, ^b^
	Louwers et al. 15	Significant	31^b^
Jebsen–Taylor Hand Function Test^27^	Van Heest et al.^19^	Not significant	Unknown^c^
House Functional Classification^23^	Carlson et al.^20^	Significant	Unknown^c^
	Libberecht et al. 18	Significant	Unknown^c^
	Roth et al.^21^	Significant	Unknown^c^
	Donadio et al.^22^	Significant	Unknown^c^
	House et al.^23^	Significant	Unknown^c^
	Matsou et al.^24^	Significant	Unknown^c^
	Matsuo et al.^25^	Significant	Unknown^c^
Measuring performance			
Assisting Hand Assessment^28^, ^29^, ^30^, ^31^	Ponten et al.^16^	Significant	56^d^
	Van Heest et al.^14^	Significant	Unknown^a^, ^d^
	Louwers et al.^15^	Significant	71^d^
SHUEE SFA^32^	Van Heest et al.^19^	Significant	Unknown^c^
	Smitherman et al.^17^	Significant	Unknown^c^
	Van Heest et al.^14^	Significant	Unknown^c^

(b)			
Outcome measures	Study	Improvement	MCID (% of participants)
COPM‐P^15^	Louwers et al.^15^	Significant	80^e^
ABILHAND(‐kids) 15	Louwers et al.^15^	Significant	55^f^
Visual Analogue Scale^15^	Louwers et al.^15^	Significant	62^g^

a Minimal clinically important differences (MCIDs) are unknown, because no individual data are given in the paper.

b The percentage with a true change (with 95% certainty) of at least six blocks per minute (measured by the same rater before and after the intervention).

c MCIDs are unknown for this outcome measure.

d The percentage with at least the smallest detectable change of five Assisting Hand Assessment units (with 95% certainty), which reflects a true change (not measurement error alone) with a standard deviation of 1.96.

e The percentage with at least a 2‐point change, which is considered to be a clinically meaningful difference between groups of patients.

f The percentage with at least the smallest detectable change of 1.18 logits (with 95% certainty), which reflects a true change (not measurement error alone) with a standard deviation of 1.96.

g The percentage with at least the MCID of 1.37cm. SHUEE SFA, Shriners Hospital Upper Extremity Evaluation spontaneous functional analysis; COPM‐P, Canadian Occupational Performance Measure performance scale.

## Supporting information


**Figure S1:** Flow diagram of studies identified for inclusion in the review.

**Table SI:** Summary of included studies

**Appendix S1** **: **Search strategy.
