# Supplementary material for: Effects of upper extremity surgery on activities and participation of children with cerebral palsy: a systematic review
Source: Dev Med Child Neurol. 2019 Jul 23;62(1):21–7. doi: 10.1111/dmcn.14315 (PMC6916411; doi:10.1111/dmcn.14315)
Supplement: Supplementary file 1 — Figure S1: Flow diagram of studies identified for inclusion in the review. [file DMCN-62-21-s001.doc]

**Figure S1. Flow diagram**

**Identification**

**Records identified through database searching**

(MEDLINE, Embase, psycINFO)
**(n=8.552)**

**Screening**

**Included**

**Eligibility**

**Records after duplicates removed**
**(n=6.501)**

**Records screened by title and abstract**

**(n=6.501)**
(n=5656)

**Records excluded (n=6.361)**

- Not in English (n=603)
- Not relevant (n=5.758)

**Full-text articles assessed for eligibility
(n =140)**

**Full-text articles excluded (n=114)** reasons:

- Outcome (n=51)
- Study design (n=19)
- Population (n=20)
- background article (n=9)
- Publication type (n=15)

**Studies included in qualitative synthesis
(n=26)**

**Studies included in quantitative synthesis
(n =12)**

**Studies included:**

**(n =12)**

**Full-text articles excluded (n=14)** reasons:

- Outcome (n=8)
- Population (n=6)
