# Supplementary material for: Effects of upper extremity surgery on activities and participation of children with cerebral palsy: a systematic review
Source: Dev Med Child Neurol. 2019 Jul 23;62(1):21–7. doi: 10.1111/dmcn.14315 (PMC6916411; doi:10.1111/dmcn.14315)
Supplement: Supplementary file 2 — Table SI: Summary of included studies [file DMCN-62-21-s002.docx]

Table SI: Summary of included studies

| Study | Study design | Patients (*n*) | CP (%) | Mean age (range) | Mean follow-up (range) | Comparative subgroups on the basis of: | Surgical (contra-)indication | Surgical technique |
| --- | --- | --- | --- | --- | --- | --- | --- | --- |
| Carlson et al.^20^ | Retrospective review | 24  8 | 100 | 10y (3–20)  14y (5–20) | 22mo (7–144)  18mo (6–51) | Surgical intervention  Fixed elbow deformity/contractures less than 45° ‘dynamic’, more than 45° ‘static’ | Indication: dynamic elbow flexion deformities more than 30°.  Contraindication: deformity is unresponsive to therapy (botulinum neurotoxin A injections and occupational therapy with bracing and casting). | Specific surgical interventions (fixed elbow deformity): lengthening biceps, brachialis, BR.  z-lengthening biceps, subtotal myotomy of the brachialis, anterior elbow capsule release, and proximal release of the brachialis. |
| Donadio et al.^22^ | Retrospective cohort study | 20 | 100 | 16y 2mo (12–17y) | 22mo (12–38) |  | Indication: contracture of the upper limb who had an arthrodesis of the wrist.  Contraindication: open growth plate. | Specific surgical interventions (wrist flexion deformity): wrist arthrodesis |
| House et al.^23^ | Retrospective review | 56 | 100 | 11y 10mo (4–20y) | 2–12y |  | Indication: deformity and imbalance of the thumb.  No contraindications. | Specific surgical interventions (thumb-in-palm deformity): lengthen AP, stripping first dorsal interosseous, lengthen FPL, PL to APL, PL+EPL, PL to EPB, BR+APL, BR+EPB, BR to EPL, FCR+APL, APL+EPL, ECRL+APL  Tenodesis: APL+FCR, EPB+FCR, EPL+FCR, EPL to EPB, Fusion: MP joint, CMC joint, IP joint |
| Libberecht et al.^18^ | Retrospective cohort study | 15 | 75 | 13y 11mo (4–30y) | 23mo (4–84) |  | No specific (contra)indications mentioned. | Specific surgical interventions (wrist flexion deformity): FCU to ECRB combined with FL, PT re-routing, PT to EPL, PT release, FAR, FL+PT release |
| Louwers et al.^15^ | Prospective cohort study | 39 | 100 | 14y 9mo (7y 10mo–19y 7mo) | 9mo (6–11) |  | Contraindications: in case of doubts about (1) the presence of dyskinesia, (2) motivation for intensive therapy, (3) achievability of the patient-specified goals, or (4) a large discrepancy between the ability to use and the actual use of the affected hand, alternative conservative treatment options were discussed with the patient, with the possibility for future re-assessment by the UES team. | Multi-level surgery: Z-lengthening biceps, brachialis muscle slide, release: PT and/or FCU, transfer: FCU . AP muscle slide combined with EPL re-routing. Fractional lengthening of the extrinsic finger flexor. Capsulodesis thumb MCP. Stabilization of Swanneck deformities in the fingers. |
| Matsou et al.^24^ | Retrospective case series | 19 | 100 | 13y (6–23) | 4y (2–7) |  | Indication: deformity and rigidity of the fingers.  No contraindications. | Specific surgical interventions (finger deformity): combined release FDP+FDS+EDC |
| Matsou et al.^25^ | Retrospective cohort study | 26 | 100 | 13y (9–35) | 4.5y (2–10y) |  | No specific (contra)indications mentioned. | Specific surgical interventions (finger deformity): combined release FDP+FDS+intrinsic muscles. |
| Ponten et al.^16^ | Prospective cohort study | 18 | 78 | 11y (6–16) | 7mo (5–14) |  | No specific (contra)indications mentioned. | Multi-level surgery: biceps lengthening, PT release, PT re-routing, FCU lengthening, FCU to EDC, FCU to ECRB, FCU to ECRL, FCR to ECRL, ECU to ECRB, BR to ECRB, PL release, finger flexor release/lengthening, thumb abduction (number of procedures) and swan neck procedures |
| Roth et al.^21^ | Retrospective cohort study | 17 | 100 | 8y 4mo (3y 4mo–15y 5mo) | 2y 7mo (11mo–4y 7mo) |  | No specific indications mentioned.  Contraindications: impaired vision and poor sensation together. | Multi-level surgery: pronator to supinator transfer, FCU to ECRB, BR to APL and EPB, Fusion MP |
| Smitherman et al.^17^ | Retrospective case–control series | 40 | 100 | 13y 1mo (6y 4mo–17y 8mo) | 14mo (7–24) |  | No specific (contra)indications mentioned, besides using the Shriners Hospital Upper Extremity Evaluation. | Multi-level surgery: FCU to ECRB, wrist and MCP arthrodesis, thumb MCP sesamoid capsulodesis, AP release, EPL re-routing, PT lengthening, fractional lengthening FDS-FDP-FPL, BB lengthening |
| Van Heest et al.^19^ | Retrospective case series | 13 | 100 | 10y 8mo (7–24y) | 3y 7mo (1–10y) |  | No specific (contra)indications mentioned. | Multi-level surgery:  FCU to ECRB, BR to ECRB, ECU to ECRB, FCU lengthening, flexor pronator slide, Wrist fusion |
| Van Heest et al.^14^ | (Pseudo-)RCT | 16 | 100 | 4–16y | 1y | Different interventions: tendon transfer surgery, botulinum neurotoxin injections, regular ongoing therapy. | Indications: standard for tendon transfer surgery: (1) pronation deformity; (2) deficient active wrist extension with adequate digital control, and wrist flexion deformity with the FCU as the primary deforming force; and (3) adduction of the thumb ray with flexion at the thumb MC) joint, with the inability to make a fist with the thumb outside the flexed digits.  Contraindication: House score of 0, previous upper-extremity surgery, upper extremity botulinum neurotoxin injections within the previous 12mo. | Multi-level surgery: FCU to ECRB, PT release, EPL re-routing+AP release |

CP, cerebral palsy; BR, brachioradialis; AP, abductor pollicis; FPL, flexor pollicis longus; PL, palmaris longus; APL, abductor pollicis longus; EPL, extensor pollicis longus; EPB, extensor pollicis brevis; FCR, flexor carpi radialis; ECRL, extensor carpi radialis longus; M(C)P, meta(carpo)phalangeal; CMC, carpometacarpal; IP, interphalangeal; FCU, flexor carpi ulnaris; ECRB, extensor carpi radialis brevis; FL, fractional lengthening of flexor tendons; PT, pronator teres; FAR, flexor aponeurotic release; FDP, flexor digitorum profundus; FDS, flexor digitorum superficialis; EDC, extensor digitorum communis; ECU, extensor carpi ulnaris; BB, biceps-brachialis.
