# Supplementary material for: Effects of upper extremity surgery on activities and participation of children with cerebral palsy: a systematic review
Source: Dev Med Child Neurol. 2019 Jul 23;62(1):21–7. doi: 10.1111/dmcn.14315 (PMC6916411; doi:10.1111/dmcn.14315)
Supplement: Supplementary file 3 — Appendix S1 : Search strategy. [file DMCN-62-21-s003.docx]

Appendix 1. Search strategy

| **Ovid MEDLINE(R) ALL <1946 to September 06, 2018>**  **Search date: 7 September 2018** | | | |
| --- | --- | --- | --- |
| **#** | **Searches** | **Results** | **After deduplication** |
| 1 | cerebral palsy/ or (exp upper extremity/ and (paralysis/ or hemiplegia/)) | 21600 |  |
| 2 | (cerebral palsy or upper limb spasticity or (brain damage and (hand adj3 function*)) or spastic hemiplegia).ab,kf,ti. | 20997 |  |
| 3 | 1 or 2 | 27984 |  |
| 4 | (lengthening or slide? or release or rerout* or stabilization or capsulodesis).ab,kf,ti. | 663909 |  |
| 5 | (surgery or surgical* or treatment or therapy or therapeut*).ab,hw,kf,ti. or th.fs. | 7581202 |  |
| 6 | (upper extremit* or upper limb* or hand? or shoulder? or elbow? or wrist? or thumb? or finger?).ab,hw,kf,ti. | 638839 |  |
| 7 | 5 and 6 | 220490 |  |
| 8 | 4 or 7 | 874662 |  |
| 9 | 3 and 8 | 3089 |  |
| 10 | remove duplicates from 9 | **3077** | **3072** |
|  |  |  |  |
| **Embase Classic+Embase <1947 to 2018 September 6> [Ovid interface]**  **Search date: 7 September 2018** | | |  |
| **#** | **Searches** | **Results** | **After deduplication** |
| **#** | **Searches** | **Results** | **After deduplication** |
| 1 | cerebral palsy/ or (exp *upper limb/ and (*paralysis/ or *hemiplegia/)) | 37272 |  |
| 2 | (cerebral palsy or upper limb spasticity or (brain damage and (hand adj3 function*)) or spastic hemiplegia).ab,kw,ti. | 30880 |  |
| 3 | 1 or 2 | 41228 |  |
| 4 | (lengthening or slide? or release or rerout* or stabilization or capsulodesis).ab,kw,ti. | 850595 |  |
| 5 | (surgery or surgical* or treatment or therapy or therapeut*).ab,hw,kw,ti. | 10732161 |  |
| 6 | ("7" or "8" or "9" or "33" or "34").ec. | 4685454 |  |
| 7 | (upper extremit* or upper limb* or hand? or shoulder? or elbow? or wrist? or thumb? or finger?).ab,hw,kw,ti. | 905263 |  |
| 8 | (5 or 6) and 7 | 476972 |  |
| 9 | 4 or 8 | 1309808 |  |
| 10 | 3 and 9 | 5048 |  |
| 11 | remove duplicates from 10 | **4960** | **3259** |
| **PsycINFO <1806 to to September Week 1 2018 (Ovid)**  **Search date: 7 September 2018** | | |  |
| **#** | **Searches** | **Results** | **After deduplication** |
| 1 | cerebral palsy/ or ((arm/ or shoulder/ or wrist/ or elbow/) and (paralysis/ or hemiplegia/ or hemiparesis/)) | 4999 |  |
| 2 | (cerebral palsy or upper limb spasticity or (brain damage and (hand adj3 function*)) or hemiplegia).ab,id,ti. | 8202 |  |
| 3 | 1 or 2 | 8495 |  |
| 4 | (lengthening or slide? or release or rerout* or stabilization or capsulodesis).ab,id,ti. | 48401 |  |
| 5 | (surgery or surgical* or treatment or therapy or therapeut*).ab,hw,id,ti. | 870006 |  |
| 6 | (upper extremit* or upper limb* or hand? or shoulder? or elbow? or wrist? or thumb? or finger?).ab,hw,id,ti. | 105475 |  |
| 7 | 5 and 6 | 16768 |  |
| 8 | 4 or 7 | 64840 |  |
| 9 | 3 and 8 | 515 |  |
| 10 | remove duplicates from 9 | **515** | **170** |
|  |  |  |  |
|  | **Totaal** | **8552** | **6501** |
